# Gene-based Genomewide Association Analysis: A Comparison Study

**DOI:** 10.2174/13892029113149990001

**Published:** 2013-06

**Authors:** Guolian Kang, Bo Jiang, Yuehua Cui

**Affiliations:** 1 Department of Biostatistics, St. Jude Children’s Research Hospital, Memphis, TN 38105;; 2 Department of Biostatistics, The University of Alabama at Birmingham, Birmingham, AL 35294;; 3 Department of Statistics and Probability, Michigan State University, East Lansing, MI 48824 USA

**Keywords:** Gene-centric, Genome-wide association study, Monte carlo, Entropy, Minimum p-value method.

## Abstract

The study of gene-based genetic associations has gained conceptual popularity recently. Biologic insight into the etiology of a complex disease can be gained by focusing on genes as testing units. Several gene-based methods (e.g., minimum p-value (or maximum test statistic) or entropy-based method) have been developed and have more power than a single nucleotide polymorphism (SNP)-based analysis. The objective of this study is to compare the performance of the entropy-based method with the minimum p-value and single SNP–based analysis and to explore their strengths and weaknesses. Simulation studies show that: 1) all three methods can reasonably control the false-positive rate; 2) the minimum p-value method outperforms the entropy-based and the single SNP–based method when only one disease-related SNP occurs within the gene; 3) the entropy-based method outperforms the other methods when there are more than two disease-related SNPs in the gene; and 4) the entropy-based method is computationally more efficient than the minimum p-value method. Application to a real data set shows that more significant genes were identified by the entropy-based method than by the other two methods.

## 1 INTRODUCTION

Single nucleotide polymorphism (SNP)–based genome-wide association studies (GWAS) have been a popular and successful method to identify disease-related SNPs. However, this approach has much lower power when the number of SNPs increases and SNPs are correlated, especially when their effect sizes are small and only their cumulative effect is associated with a disease. Gene- or region-based analysis may have higher power to identify the causal variants that affect the complex disease, because it takes into consideration the correlations among SNPs within a single gene.

The simplest method for gene-based analysis is the SNP-based method, in which each genotyped SNP is tested for association, and multiple testing corrections based on the Bonferroni procedure are applied to control the type-I error rate. The most widely used single SNP-based association test method is Cochran-Armitage trend test (CATT) which has high power under additive and multiplicative disease models but much low power under recessive disease model [1-4]. The genotypic test based on a 2×3 contingency table is robust to different disease models [5]. Some other innovative methods include entropy-based method which is generally as good as or even more powerful than the genotypic test [5, 6]. The SNP-based method for gene-based analysis has low power when the causal variants are highly correlated with one or more genotyped SNPs and when the causal SNPs are not genotyped. The power of the SNP-based method can be improved by combining the information from neighboring SNPs within a single gene. Several methods have been developed to analyze multiple SNPs within the same gene simultaneously. These methods include Fisher’s method for combining p-values by a logarithm function of p-values and the minP (minimum p-value) or maxT (maximum test statistics) method in which the significance level can be determined by the observed p-value. However, the empirical p-value must be calculated by using permutation, because the limiting distributions of Fisher’s statistic and minP (maxT) statistic are unknown under the null hypothesis that the gene is not associated with the disease. 

Another alternative method to combine multiple SNPs is to do multivariate tests. Chapman and Whittaker proposed a multivariate score test statistic that is equivalent to the score test for the logistic regression model [7]. Another test statistic based on an empirical Bayesian model for the parameters was similar to the above multivariate score test statistic [8]. Wang and Elston proposed a test statistic using a weighted Fourier transform of the genotypes to reduce the test degrees of freedom [9]. Chapman and Whittaker compared the above five methods by simulation studies, and they found that the minP (maxT) and Goeman’s method perform well over a range of scenarios [7]. 

For the minP (maxT) method, a Monte Carlo (MC) method can be used to evaluate the empirical p-values based on approximating the joint distribution of the test statistics by an MC-sampling approach. This is computationally feasible compared with a permutation method [10]. An entropy-based test statistic was recently proposed to test gene-disease association based on the joint genotypes on multiple SNPs within a gene and a cluster-based analysis method was used to reduce the degrees of freedom of the test statistic [11]. 

In this study, we compare three methods, namely the single SNP-based method, the maxT method with MC sampling to estimate the empirical p-value, and an entropy-based method, by simulation studies and real data analysis. We start with a detailed description of each method, followed by simulations and real data analysis. 

## 2 METHODS

### 2.1 MaxT (or minP) Method with Monte Carlo Sampling

Much of what follows in the section below is adapted from Lin [10]. Consider one gene with *m* genic SNPs, each with two alleles. Let *Y_i_* be the phenotypic value of the *i*-th individual; let *X _ji_* = 0, 1, or 2 be the genotype of *i*-th individual at locus *j*; and let $\bar{Y}=\sum_{i=1}^{n}Y_{i}/n$ and ${\bar{X}}_{j}=\sum_{i=1}^{n}X_{\mathrm{ji}}/n$ , where 1 ≤ *i* ≤ *n*, 1 ≤ *j* ≤ *m*, and *n* is the sample size. The test statistic for the *j*-th locus within this gene is defined as $T_{j}=U_{j}^{T}V_{j}^{-1}U_{j}$ and *j*=1, 2,…, m, where $U_{j}=\sum_{i=1}^{n}U_{\mathrm{ji}},$ , $U_{\mathrm{ji}}=\left(Y_{i}-\bar{Y}\right)X_{\mathrm{ji}}$, and $V_{j}=\sum_{i=1}^{n}U_{\mathrm{ji}}U_{\mathrm{ji}}^{T}$. This test statistic follows an *χ* ^2^ distribution with *r _j_* degrees of freedom, where *r _j_* is the dimension of *U _j_*. 

The test statistics (*T_1_, T_2_,…, T_m_*) may be correlated due to linkage disequilibrium among SNPs within one gene. The p-values evaluated by using the actual joint distribution of (*T_1_, T_2_,…,T_m_*) can be computationally intensive. Lin [10] proposed an MC method to approximate the actual joint distribution to evaluate the empirical p-values by MC sampling. The MC method defines ${\tilde{T}}_{j}={\tilde{U}}_{j}^{T}V_{j}^{-1}{\tilde{U}}_{j}$ , where ${\tilde{U}}_{j}=\sum_{i=1}^{n}U_{\mathrm{ji}}G_{i}$, and *G_1_, G_2_,…,G_n_* are independent, standard, normal, random variables that are independent of the data. The method then uses the joint distribution of ${\tilde{T}}_{j}s$ to approximate the joint distribution of *T_j_*s on the basis of obtaining realizations from distributions of ${\tilde{T}}_{j}s$ by repeatedly generating the normal random samples *G_1_, G_2_,…,G_n_*. Let ($t_{1},t_{2},\cdot \cdot \cdot ,t_{m}$) be the observed values of the test statistics (*T_1_, T_2_,…, T_m_*), and let $t_{\max}=\max\left\{t_{1},t_{2},\cdot \cdot \cdot ,t_{m}\right\}$. If $\mathrm{Pr}\left({\tilde{T}}_{\max}\geq t_{\max}\right)<\alpha$ , where *α* is the preset significance level, then the null hypothesis that this gene is not associated with the disease is rejected.

### 2.2 Entropy-based Test Statistic and Genotype Grouping via Penalized Entropy

For one gene with *m* genic SNPs, there is a total of 3^*m*^ joint genotypes. However, the real number of joint genotypes is much less. Denote the number of observed joint genotypes for one gene by *s* (*s*<3^*m*^). Let $p_{i}^{A}$ and $p_{i}^{U}$ (1 ≤ *i* ≤ *s* ) be the frequencies of the *i*-th joint genotype in cases and controls, respectively. Then the entropy-based test statistic for testing the association between this gene and a disease is as follows [11]:

(1) $$T^{\mathrm{gene}}=\left(S^{A}-S^{U}\right)W^{-1}{\left(S^{A}-S^{U}\right)}^{T},$$

where $S^{A/U}=\left[-p_{1}^{A/U}\log\left(p_{1}^{A/U}\right),\cdot \cdot \cdot ,-p_{m}^{A/U}\log\left(p_{m}^{A/U}\right)\right]$, W= *D^A^*Σ*^A^D^A^* / *n^A^* + *D^U^*Σ*^U^D^U^* / *n^U^*, *n^A^*^/*U*^ is the number of cases and controls, and 
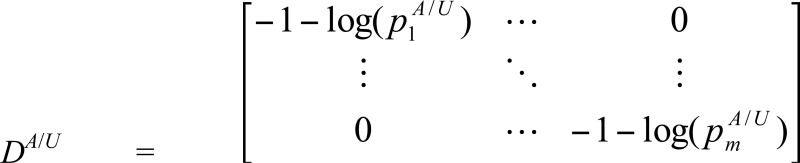
 , 
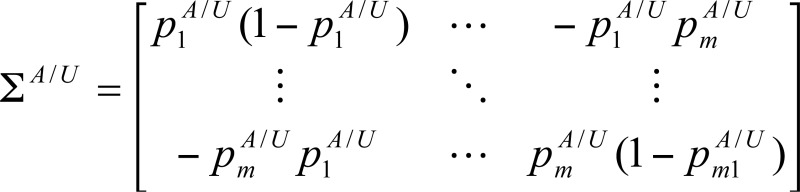
 .

Under the null hypothesis that there is no association between this gene and a disease, *T*^gene^ follows a central *χ* ^2^ distribution with *m*-1 degrees of freedom.

When the number of genic SNPs is high, the degree of freedom increases so that the power will decrease. To increase the power, the rare joint genotypes could be grouped into common ones by using the penalized entropy measure (PEM) [11]: $I=-\left(\sum_{j=1}^{k}p_{j}\log_{2}p_{j}\right)-2\log_{2}k/m_{k}$ , where *m_k_* is the number of *k*-th joint genotypes. The joint genotype set with maximum value of *I *will be the corresponding common joint genotype. To do so, we first sort all joint genotypes in descending order, according to their frequencies. Then we calculate the PEM by adding one joint genotype to the present joint genotype set. If the PEM begins to decrease when the k-th joint genotype is added to the current set, the common joint genotype set will include the former k-1 joint genotypes.

Once the grouping threshold is determined, we can proceed to calculate the similarities between one rare-joint genotype with frequency less than the threshold and all common genotypes and then group it with the common one that is the most similar. 

## 3 SIMULATION STUDIES

We evaluated the performance of the three methods described above by using simulation studies. We simulated case-control samples in two methods: one using a linkage-disequilibrium (LD)-based method similar to methods in [10, 11], and the other using an MS program developed by Hudson [12] that is similar to programs developed by Tzeng [13]. Although we will not discuss the LD-based simulation method here (see [11]), we describe below the detailed process to generate samples by the MS program.

### 3.1 MS Program

We used the MS program developed by Hudson [12] to simulate haplotypes for each individual to form individual genotype data. The main parameters under the coalescent model for generating haplotypes were set as: the effective diploid population size *n_e_* is 1×10^4^ ; the scaled recombination rate for the whole region of interest, 4*n_e_γ* / *bp* , is 4 ×10^-3^ , where the parameter *g* is the probability of crossover per generation between the ends of the haplotype locus being simulated; the scaled mutation rate for the simulated haplotype region, 4*n_e_µ* / *bp* , is set to be 5.6 × 10^-4^ for the region of simulated haplotypes; and the length of sequence within the region of simulated haplotypes, *n* sites, is 10 kb. Similar parameter settings can be found in other studies [10, 12, 13]. We set the number of SNP sequences in the simulated sample to 100 for each gene and run the MS program to generate the haplotype sample on the basis of these parameter settings. Then we randomly selected a segment of 10 adjacent SNPs as a haplotype. The two haplotypes are randomly drawn from the simulated sample containing 100 10-SNP haplotypes and are paired to form an individual genotype.

### 3.2 Phenotype Simulation

In reality, we do not know the true functional mechanism for a given gene, so it is difficult to simulate the true functional variants and the true functional mechanism within a gene [13]. Here, we considered three scenarios to mimic the situation of a complex disease in which there is one, two, or three disease-related SNPs within a given gene. For cases with two or three disease-related SNPs, complex interactions occur among the SNPs. Here we briefly illustrate how the disease phenotypes are simulated.

#### Scenario 1.

Let *f_0_*, *f_1_*, *f_2_* be three penetrances of three genotypes. Denote λ_1_ = *f*_1_/*f*_0_, λ_2_ = *f*_2_/*f*_1_as the genotype-relative risks (GRRs). Let *p* be the disease allele frequency, and denote the disease prevalence as *k*. Then the three penetrances can be calculated for an additive, dominant, or recessive disease model (Table **1**). We omit a multiplicative model, because the results of that model are similar to those from the additive model. Once *f* is determined, the case/control status is simulated according to a Bernoulli distribution, with the probability of success *f *conditional on the observed genotype data.

For a disease model with two or three interactions of disease-related SNPs within a single gene (Scenarios 2 and 3), we follow the cases given in [14].

#### Scenario 2.

For the two-locus-interaction disease model, we denote the two-locus genotypes as $\left(G_{A},G_{B}\right)\in {\left(0,1,2\right)}^{2}$, which represents the number of risk alleles at each disease-related SNP A and B. The two-locus-interaction disease model is as follows:

Model 1: Odds(G_A_, G_B_) = γ(1+θ)*G_A_* +*G_B_*

Model 2: Odds(G_A_, G_B_) = γ(1+θ)*G_A_I* (*G_A_* >0)+*G_B_I* (*G_B_* >0)

Model 3: Odds(G_A_, G_B_) = γ(1+θ)*I* (*G_A_* >0∩*G_B_* >0)

where *λ* is the baseline effect, and *θ* is the genotypic effect.

#### Scenario 3.

For the three-locus-interaction disease model, we denote the three-locus genotypes as $\left(G_{A},G_{B},G_{C}\right)\in {\left(0,1,2\right)}^{3}$, which represents the number of risk alleles at each disease-related SNP A, B, and C. The three-locus-interaction disease model is as follows:

Model 1: Odds(G_A_, G_B_, G_C_) = γ(1+θ)*G_A_* +*G_B_* +*G_C_*

Model 2: Odds(G_A_, G_B_, G_C_) = γ(1+θ)*G_A_I* (*G_A_* >0)+*G_B_I* (*G_B_* >0)+*G_C_I* (*G_C_* >0)

Model 3: Odds(G_A_, G_B_, G_C_) = γ(1+θ)*I* (*G_A_* >0∩*G_B_* >0∩*G_C_* >0)

where *λ* and *θ* are the same as in Scenario 2. Once the disease-related SNPs are determined, the case-control status can then be simulated according to a multinomial distribution conditional on the observed genotype data.

We simulated data sets with 400 cases and 400 controls or 800 cases and 800 controls. For the evaluation of type one error rate, we simulated data sets using both LD-based and MS methods but for power, we only used MS method because it can better mimic the biological data. For each data set, we applied the three methods described above. The type-I error rate was estimated based on 1000 replicates, and the power was estimated based on 100 replicates at a significance level of 0.05. For the maxT method, the empirical p-value was obtained based on 10,000 normal samples.

## 4 REAL DATA ANALYSIS

To compare the three methods, we applied them to a large-scale, candidate-gene study. The data set contains 225 cases and 585 controls on 190 candidate genes in a genetic association study of preeclampsia [15]. We removed SNPs with minor allele frequencies less than 0.05 and focused on the remaining 819 SNPs. We also removed 27 genes carrying only one SNP. Similar to [11], we used a nominal level of 0.005 for the gene-based method and 0.005 dividing the number of SNPs within each gene for SNP-based method. 

(Table **2**) lists the p-values of significant genes and SNPs for the three methods. The genes and SNPs that showed significant effects are formatted in bold. The entropy-based method identified seven significant genes among the 190 genes evaluated. The single SNP-based method identified three significant genes, and the maxT method identified one significant gene. Thus, the gene-based entropy method identified the most number of significant genes.

## 5 SIMULATION RESULTS

(Table **3**) presents the empirical type-I error rates of the single-SNP, maxT, and entropy-based methods based on the MS program and LD-based method. From (Table **3**), we see that the maxT and entropy-based methods control the type-I error rate quite well. The latter also controls as the sample size increases. However, the single-SNP method has a much lower type-I error rate, which means that this method may have lower power. We also simulated 10 SNPs with *r*^2^=0.9, 0.5, and 0 within one gene by using the LD-based method and found that all three methods control the type-I error rate well.

(Table **4**) presents the estimated power of the SNP-based, maxT, and entropy-based methods for one disease-related SNP within a single gene. The maxT method appeared to be the most powerful among the three methods. The entropy-based method had lower power than the maxT method, because when one disease-related SNP occurs within a gene, the cluster number in the entropy-based method will be large, so that the degree of freedom of the test statistic in equation (1) is high. This will affect the power of the entropy-based method. 

(Tables **5** and **6**) present the estimated power of the three methods for situations in which two or three disease-related SNPs occur within a single gene. The entropy-based method appeared to be the most powerful method, and the single SNP–based method was the least powerful. This makes sense because when there are two or three interacting-disease-related SNPs within one gene, the cluster number of the observed joint genotypes will be small. Thus, the degrees of freedom of the test statistic in equation (1) will be small, which will improve the power of the entropy-based method.

## 6 DISCUSSION

We have compared three gene-based association approaches by conducting simulation studies and one real data set analysis. Simulation results show that 1) all three methods effectively control the type-I error rate; 2) the single SNP–based method is very conservative; 3) when there is one disease-related SNP within a gene, the maxT method is the most powerful; 4) when there are two or three disease-related SNPs within a gene, the entropy-based method is the most powerful. Real data analysis shows that the entropy-based method identifies more significant genes than do the other two methods. In addition, we have compared the computing time used by the three methods and found that the entropy-based method is computationally more efficient than the maxT method. 

Given the unknown number of causal SNPs as well as the complex structure among/between causal and non-causal SNPs within the gene, and the complex underlying disease gene actions, the relative performance of different approaches for gene-based association tests strongly depends on different realistic scenarios. Considering genes as testing units, sometimes we have to move forward to pursue gene-based interactions to get better biological insights into the etiology of complex diseases [16]. As new approaches are increasingly developed, we believe that no single approach is universally superb to others [4]. We suggest that users explore as many different approaches as possible and choose the best one based on their biological experience. 

Rare variants may play an important role to explain the missing heritability of complex disease in post-GWAS research. The correlations between rare and common SNPs and among rare variants are generally weak [17], and the number of causal rare SNPs each with moderate or large effect sizes may be large [18]. The novel statistical or computational methodologies for analyzing rare variants focusing on genes are urgently needed with the availability of large scale exome or wholegenome sequencing data [19]. The relative performance of these approaches for gene-based association tests is worthy of further investigation.

## Figures and Tables

**Table 1.** Single-SNP Disease Model

Disease Model	*f*_0_ a	*f*_1_	*f*_2_
Additive	*prev* /(1– 2 *p* + 2 *pλ*) b	*λ f_0_*	(2 *λ*-1)*f_0_*
Dominant	*prev* /((1 – *p*) ^2^ + *λp*(2-*p*))	*λ f_0_*	*λ f_0_*
Recessive	*prev* /(1 + *p* ^2^ *λ*^2^ – *p* ^2^)	*f_0_*	*λ f_0_*

a The *f*_0_, *f*_1_, *f*_2_ are three penetrances of genotypes.

b In additive and dominant models, λ = λ_1_, and in a recessive model, λ = λ_2_.

**Table 2.** Analysis of the Preeclampsia Data Set Using the SNP-Based, Gene-based Entropy, and MaxT Methods

Gene (No. of SNPs)	maxT^a^	Entropy^b^	SNP^c^	SNP-based Method
*APOB* (9)	0.0379	**0.0015^d^**	rs5456814	0.0165
*F13B* (4)	0.0282	**0.0029**	rs28787657	**0.0010**
*F2* (7)	0.5812	**0.0020**	rs28886771	0.0021
*FGF4* (3)	**0.0047**	**0.0039**	rs634043464	0.0067
*IGF2R* (14)	0.7919	**0.0005**	rs41410456	0.0330
*MMP10* (8)	0.1150	**0.0006**	rs634850223	0.0280
*PDGFC* (2)	0.0527	**0.0036**	rs634820282	0.032
*IGF1R* (7)	0.1312	0.1902	rs40893937	**0.0006**
*NOS2A* (10)	0.3695	0.0547	rs9678181	**0.0001**

a Data were obtained using the maximum test statistic method.

b Data were obtained using the entropy-based method.

c Only SNPs with the smallest P-values within the corresponding genes are listed.

d Bold formatting of data indicates significant p-values.

**Table 3.** The Estimated Type I Error Rate Under the Null Hypothesis of No Association by Using MS Program

SS	MS Program			LD-based Programs								
				*r* ^2 ^= 0.9			*r* ^2 ^= 0.5			*r* ^2 ^= 0.0		
	maxT^a^	Entropy^b^	SNP^c^	maxT	Entropy	SNP	maxT	Entropy	SNP	maxT	Entropy	SNP
400	0.05	0.06	0.03	0.05	0.06	0.027	0.06	0.06	0.06	0.04	0.04	0.04
800	0.05	0.05	0.02	0.04	0.06	0.019	0.05	0.06	0.04	0.06	0.05	0.05

a SS, sample size.

b Data were obtained using the maximum test statistic method.

c Data were obtained using the entropy-based method.

d Data were obtained using the single-SNP–based method.

**Table 4.** The Estimated Power of Gene-based Association Tests, Assuming One Disease-related SNP Occurs Within the Gene, Under Different Sample Sizes and Different Disease Models

Disease Model	GRR^a^	N=400			N=800			
		maxT^b^	Entropy^c^	SNP^d^		maxT	Entropy	SNP
Additive	1.4	1	0.56	0.60		0.95	0.92	0.94
	1.6	1	0.91	0.955		1	1	1
	1.8	1	0.975	0.990		1	1	1
Dominant	1.4	0.47	0.39	0.36		0.65	0.62	0.74
	1.6	0.75	0.65	0.73		0.94	0.90	0.95
	1.8	0.88	0.89	0.90		0.99	0.99	0.99
Recessive	1.4	0.22	0.26	0.20		0.29	0.29	0.37
	1.6	0.32	0.34	0.34		0.64	0.74	0.77
	1.8	0.54	0.63	0.59		0.86	0.92	0.98

a GRR, genotype relative risks.

b Data were obtained using the maximum test statistic method.

c Data were obtained using the entropy-based method.

d Data were obtained using the single-SNP–based method.

**Table 5.** The Estimated Power of Gene-based Association Tests, Assuming that Two Disease-related SNPs Occur Within a Gene, Under Different Sample Sizes and Different Disease Models

Disease Model	(BL,GE) a	N=400			N=800		
		maxT^b^	Entropy^c^	SNP^d^	maxT	Entropy	SNP
Model 1	(1,0.5)	0.31	0.42	0.19	0.61	0.76	0.37
	(1,0.7)	0.54	0.71	0.35	0.87	0.93	0.72
	(1,0.9)	0.78	0.89	0.61	0.99	1	0.96
Model 2	(1,0.5)	0.20	0.29	0.19	0.52	0.54	0.49
	(1,0.7)	0.34	0.45	0.38	0.66	0.77	0.79
	(1,0.9)	0.52	0.65	0.59	0.90	0.96	0.97
Model 3	(1,0.5)	0.17	0.25	0.10	0.51	0.49	0.54
	(1,0.7)	0.43	0.56	0.43	0.66	0.77	0.76
	(1,0.9)	0.41	0.59	0.50	0.84	0.91	0.92

a BL, the baseline effect; GE, is the genotypic effect.

b Data were obtained using the maximum test statistic method.

c Data were obtained using the entropy-based method.

d Data were obtained using the single-SNP–based method.

**Table 6.** The Estimated Power of Gene-based Association Tests, Assuming Three Disease-related SNPs Occur Within a Gene, Under Different Sample Sizes and Different Disease Models

Disease Model	(BL,GE)^a^	N=400			N=800		
		maxT^b^	Entropy^c^	SNP^d^	maxT	Entropy	SNP
Model 1	(1,0.5)	0.54	0.56	0.42	0.92	0.88	0.81
	(1,0.7)	0.87	0.77	0.63	1	1	1
	(1,0.9)	0.95	0.94	0.87	1	1	1
Model 2	(1,0.5)	0.56	0.50	0.33	0.94	0.91	0.81
	(1,0.7)	0.87	0.76	0.73	1	0.99	0.99
	(1,0.9)	0.96	0.96	0.91	1	1	1
Model 3	(1,0.5)	0.06	0.05	0	0.01	0.08	0.03
	(1,0.7)	0.08	0.13	0.05	0.06	0.16	0.02
	(1,0.9)	0.04	0.19	0.03	0.05	0.20	0.05

a BL, the baseline effect; GE, is the genotypic effect.

b Data were obtained using the maximum test statistic method.

c Data were obtained using the entropy-based method.

d Data were obtained using the single-SNP–based method.
